# Research on the Influencing Factors of Sustainable Supply Chain Development of Agri-Food Products Based on Cross-Border Live-Streaming E-Commerce in China

**DOI:** 10.3390/foods12173323

**Published:** 2023-09-04

**Authors:** Gaofeng Wang, Zihao Zhang, Shuai Li, Changhoon Shin

**Affiliations:** 1 School of Management, Henan University of Technology, Zhengzhou 450001, China; 2 School of Economics and Management, China University of Mining and Technology, Xuzhou 221116, China; 3 College of Ocean Science and Engineering, Korea Maritime & Ocean University, Busan 49112, Republic of Korea

**Keywords:** live streaming e-commerce, cross-border agri-food products, sustainable supply chain, grounded theory, influencing factors

## Abstract

The organization and coordination of cross-border e-commerce platforms in agricultural product trading are continuously increasing, and the involvement of digital platforms has driven the integration and development of cross-border agricultural product supply chains with live-streaming e-commerce, effectively facilitating the comprehensive development of producers, sellers, and professional service providers within the ecosystem. However, despite the growing importance of this integration model in the market, there are still numerous unresolved issues from a supply chain perspective, and existing research provides relatively limited guidance on the effective operation of this integrated supply chain model. To address this gap in theoretical research, this study first delves into the essence and fundamental characteristics of sustainable cross-border agricultural product supply chains in the context of live streaming. Secondly, employing grounded theory as the primary research method and combining platform theory and ecosystem theory, an influencing factor system and an impact model for the development of sustainable cross-border agricultural product supply chains in the context of live streaming are constructed to gain a more comprehensive understanding of the complexity of this integrated supply chain model. Finally, from the perspectives of government agencies and practitioners, development pathways for sustainable cross-border agricultural product supply chains in the context of live-streaming e-commerce are proposed, aiming to enhance existing research and provide decision-making support for relevant stakeholders in formulating development strategies. The findings of this study contribute to expanding the research perspective on the ecosystem of live-streaming e-commerce and sustainable cross-border agricultural product supply chains, providing theoretical support for the establishment and operation of sustainable cross-border agricultural product supply chains in the context of live streaming. Additionally, it offers important references for promoting the sustainable development of live-streaming e-commerce and cross-border agricultural product supply chains, facilitating industry upgrading, global agricultural trade, and achieving mutually beneficial outcomes.

## 1. Introduction

In the wave of global digital innovation, the digital economy has deeply integrated with traditional trade, and digital trade has become a new engine, new momentum, and new advantage for global trade [1]. As the world’s largest producer and consumer of agricultural products, China’s “Digital Silk Road” initiative, advocated under the Regional Comprehensive Economic Partnership (RCEP) and the Belt and Road Initiative, will effectively enhance the digital infrastructure level of countries along the routes, especially developing countries, and promote the digital transformation of agriculture-related enterprises and the integration of agricultural industry [2]. Digital trade of agricultural products is deeply embedded in the global trade network. Moreover, the COVID-19 pandemic may further strengthen these trends. In the post-pandemic era, the global trade environment and geopolitical landscape have become more complex and uncertain, leading to a sharp rise in internal and external uncertainties and risks for agricultural supply chains [3,4]. In response to these influences, large-scale foreign trade enterprises have accelerated the deployment of digital online channels and shifted their strategic focus from the demand side to the supply side, promoting the emergence of new forms of integration in agricultural supply chains [5].

Live-streaming e-commerce, as an innovative form of digital trade, has become a popular choice for online shopping among domestic and international consumers due to its visual display, strong language expression, and real-time interactive experience. It has also become a new channel for Chinese agricultural products to expand into overseas markets [6]. In recent years, China has become the world’s largest market for live-streaming e-commerce [7]. According to the “China Internet Development Status Statistical Report” released in September 2022, as of June 2022, China’s internet users reached 1.051 billion, with 762 million being online live-streaming users, accounting for 68.1% of the total internet users. At the same time, using live streaming to assist in poverty alleviation and support agriculture has become an important trend in the field of Chinese e-commerce [8]. For example, leveraging the China (Tianshui) Cross-border E-commerce Comprehensive Pilot Zone, the city of Tianshui in Gansu province has organized multiple cross-border live streaming sales events for its agricultural specialties, such as Huanuo apples, Qinan peaches, cherries, and Chinese medicinal herbs. Through Alibaba’s international platform, these agricultural products from Tianshui have successfully entered foreign markets, including Malaysia and Singapore. Live-streaming e-commerce not only provides more visibility and exposure opportunities for farmers and consumers but also promotes a deeper division of labor, transaction governance, and increasing returns to scale in the industry chain, providing new ideas for revitalizing rural industries and strengthening sustainable agriculture [9]. However, the cross-border agricultural live-streaming e-commerce industry also faces a series of challenges, such as insufficient operational capacity of live-streaming platforms, discontinuity in the upstream and downstream supply chains, and industry credit crisis. Particularly, the environmental and climate impacts of this industry are gradually becoming apparent, and “sustainable development” has become an important issue in the development of cross-border agricultural live-streaming e-commerce [10]. Against the backdrop of the global rise of live-streaming e-commerce and the government’s “dual-carbon” strategy, researching the sustainable supply chain of cross-border live-streaming e-commerce for agricultural products is of practical and strategic significance.

According to related studies, domestic and international scholars have mostly focused on traditional agricultural supply chains, including agricultural supply chains [11,12,13,14,15,16], cross-border agricultural supply chains [17,18,19,20,21,22], and sustainable agricultural supply chains [23,24,25,26,27,28,29]. However, the definition and scope of the emerging supply chain model of cross-border sustainable supply chains in the context of live-streaming e-commerce have not yet been defined. Moreover, existing research methods mainly adopt quantitative empirical approaches [30,31], neglecting qualitative research based on specific case materials. Especially in the field of live-streaming e-commerce, it is difficult to obtain relevant data solely through quantitative measurement, and there is a lack of relevant theoretical frameworks. Additionally, existing research mainly focuses on a single subject or component of the supply chain, such as a specific entity [31,32,33,34,35,36,37], a specific link in the supply chain [38,39,40,41], or a specific subsystem of the supply chain [16,42,43], lacking a systematic analysis of the entire supply chain. However, the cross-border agricultural supply chain in live-streaming e-commerce is a complex open system composed of multiple subsystems [44]. The interaction between subsystems at various levels and the interaction between the system and the external environment all affect the sustainable development of the giant system. Therefore, it is necessary to study and analyze the influencing factors of the supply chain as a whole in a systematic way to fully understand and reveal its operating mechanisms and development patterns. Based on this, this study proposes the following three questions:

(1) What are the connotations and characteristics of the sustainable supply chain for cross-border agricultural products in the context of live-streaming e-commerce?
(2) What are the influencing factors for the development of sustainable supply chains for cross-border agricultural products in the context of live-streaming e-commerce?
(3) What is the mechanism of interaction among the influencing factors for the development of sustainable supply chains for cross-border agricultural products in the context of live-streaming e-commerce?

This study fills the theoretical gap in the integration of live-streaming e-commerce ecology and cross-border agricultural supply chains by exploring the connotations and characteristics of sustainable supply chains for cross-border agricultural products in the context of live-streaming e-commerce. Furthermore, a new theoretical framework is constructed based on platform theory and ecosystem theory, expanding the scope and application scenarios of relevant theories. The grounded theory approach is adopted to provide a theoretical analysis framework for the long-term operation of cross-border agricultural live streaming supply chains and enrich the relevant literature. Finally, targeted development strategies are formulated based on the interaction model of influencing factors to provide decision support for government departments and practitioners, and to offer China’s solutions and wisdom for achieving the United Nations’ 2030 Sustainable Development Goals of “Zero Hunger, Food Security, Improved Nutrition, and Promoting Sustainable Agriculture” on a global scale [45].

The rest of this study is organized as follows: Section 2 defines the connotations and characteristics of relevant concepts and introduces the theoretical foundation. Section 3 presents the research design and model construction. Section 4 reports the research results and conducts discussions. Section 5 concludes with future prospects.

## 2. Concept Definition and Theoretical Foundation

Before exploring the factors and mechanisms influencing the development of sustainable supply chains for cross-border agricultural products in the context of live streaming, we reviewed relevant theoretical domains to provide a priori theoretical sensitivity [46,47]. Due to the composite nature of this supply chain concept, which evolves from the coupling of multiple concepts and is embedded within aggregated systems across enterprises and industries, our investigation begins by considering multiple related clusters of supply chain nodes.

### 2.1. Concept Definition and Basic Characteristics

**Figure 1:**
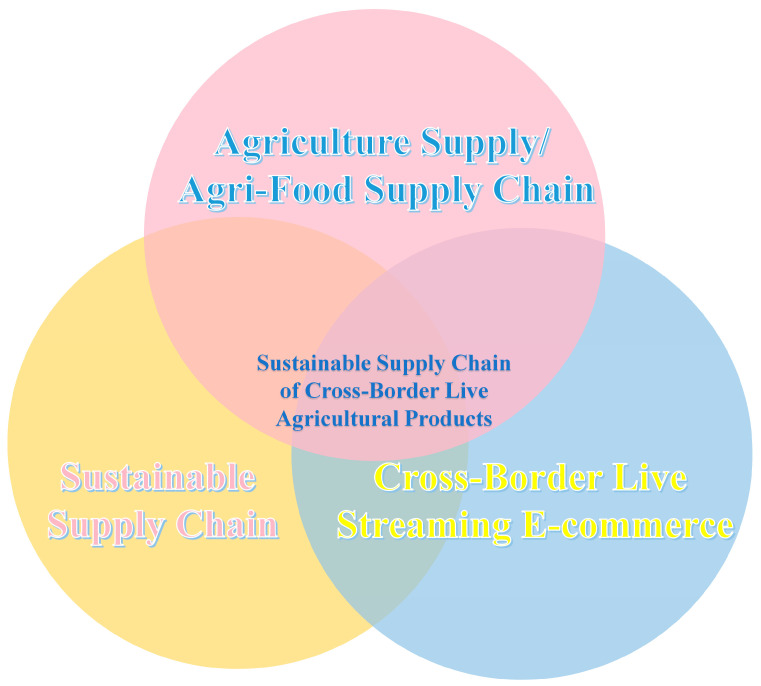
Conceptual Evolution Diagram.

The sustainable supply chain for cross-border agricultural products in the context of live streaming initially evolved from the agricultural product supply chain, inheriting its essential characteristics. With the development of cross-border trade and intensified competition among enterprises, the boundaries of the agricultural product supply chain have extended, and competition among enterprises has increasingly focused on the entire supply chain ecosystem. Sustainability has become a crucial indicator for measuring the level of development. This has given rise to two important research directions: cross-border agricultural product supply chains and sustainable supply chains. Based on these three concepts, combined with the emerging model of live-streaming e-commerce, the sustainable supply chain for cross-border agricultural products in the context of live streaming has emerged, as shown in Figure 1.

Therefore, based on the analysis and comparison of relevant literature on agricultural product supply chains [11,12,13,14,15,16], cross-border agricultural product supply chains [17,18,19,20,21,22], and sustainable agricultural product supply chains [23,24,25,26,27,28,29], this study attempts to define the connotations and characteristics of the concept of sustainable supply chains for cross-border agricultural products in the context of live streaming. The specific summary process is presented in Table 1.

The sustainable supply chain for cross-border agricultural products in the context of live streaming is a significant outcome and manifestation of the deep integration and collaborative development between the emerging model of live-streaming e-commerce and the traditional agricultural product supply chain. It relies on core enterprises engaged in agriculture and foreign trade, leveraging modern information technology to enable a supply chain system supported by live-streaming e-commerce channels, driven by real demand, and enhanced through socialized services. By controlling the flow of funds, materials, and information, this system maximizes the overall benefits of the supply chain and achieves multidimensional, sustainable, and coordinated development. In the era of implementing the new development philosophy, the cross-border sustainable supply chain for agricultural products, which carries the genes of the digital economy, exhibits four distinct characteristics.

First, the sustainable supply chain for cross-border agricultural products in the context of live streaming revolves around data as a crucial production factor. In the current era of the digitally interconnected economy, data possesses attributes of replicability, shareability, infinite growth, and infinite supply, forming an economic development model with increasing returns [48]. Empowered by the live-streaming e-commerce ecosystem, various units within the agricultural product supply chain can achieve real-time, online sharing of data and information related to production, processing, distribution, and trade. The digitized platforms built by core enterprises act as gathering grounds for data assets, facilitating continuous and automated integration, structuring, and analysis of data. Through socialized and intelligent matching services, search costs, quality control costs, information costs, transaction costs, and other expenses can be significantly reduced. This provides a strong impetus for supply chain innovation, capital activation, talent cultivation, and industrial upgrading [49].

Second, the sustainable supply chain for cross-border agricultural products in the context of live streaming connects consumer groups through live-streaming e-commerce channels, meeting their multidimensional and multilevel demands. Distinguished from conventional agricultural product supply chains, the empowerment of live-streaming e-commerce reconstructs the upstream channels of agricultural products, forming a three-dimensional framework of “people, goods, and scenes” that transcends traditional channels. At the consumer end, it integrates shopping and emotional demands, creating immersive commercial scenes [50]. This promotes the dynamic matching between high-quality products and services and consumer demands through efficient upstream channels. It satisfies consumer groups’ integrated needs in shopping, entertainment, socialization, as well as their multilevel demands for personalized, high-quality agricultural products and related services amid the backdrop of upgraded nutritional requirements.

Third, the sustainable supply chain for cross-border agricultural products in the context of live streaming is a comprehensive system engineering encompassing economic, social, environmental, and resilience aspects. This sustainable supply system covers all stages of production, distribution, processing, sales, and disposal (waste and spoilage) within the broader agricultural domain. Under the data-driven approach, it forms a closed-loop network architecture. While pursuing the maximization of supply chain benefits, it is also aligned with broader economic, social, and environmental goals. Furthermore, in the current global market environment characterized by instability, uncertainty, complexity, and ambiguity, there is a growing need for supply ecosystems to become more resilient, enhancing cross-border cooperative capacities for disaster prevention, mitigation, and response.

Finally, the sustainable supply chain for cross-border agricultural products in the context of live streaming represents an industrial ecosystem characterized by ubiquity, intelligence, and integration. Supported by modern information technologies such as cloud computing, the Internet of Things, big data, blockchain, robotics, and artificial intelligence, it enables real-time interconnection, deep integration, and collaborative development among different organizations, industry clusters, and business models within the traditional agricultural industry system. On the one hand, it profoundly transforms the production methods and organizational forms of traditional agricultural industries, developing into a data-driven, intelligent, agile, and self-connected system [51]. On the other hand, it establishes a comprehensive, multilevel, and integrated industry service system that includes logistics, finance, and consulting, helping break information barriers among different nodes of the supply chain and facilitating orderly resource exchange and sharing.

It can be observed that the sustainable supply chain for cross-border agricultural products in the context of live streaming originates from the concept of the agricultural product supply chain. It incorporates the essence of cross-border agricultural product supply chains, characterized by reliance on core enterprises and multiple actors and elements. It also assimilates the economic, social, environmental, and resilience dimensions of sustainable agricultural product supply chains. However, it has also given rise to new connotations and features within the wave of digitization, such as changes in key production factors and distribution channels. In order to further construct the theoretical support for this open and complex system, which encompasses broad scopes, multiple stakeholders, and intricate processes, we will briefly turn to the literature on applied ecosystem theory and platform theory.

### 2.2. Theoretical Foundation

The concept of an ecosystem originates from biology and refers to the interconnectedness of various organisms and their interactions with the environment through material cycles and energy conversions within a specific spatio-temporal dimension. As the basic functional unit for the flow of matter and energy and the interaction between organisms and the environment, an ecosystem is a complex entity with self-regulating capabilities [52]. Moore [53] first applied the ecosystem theory to the field of technological innovation and introduced the concept of an innovation ecosystem. The introduction of the ecosystem concept broke the limitations of traditional resource-based perspectives in the study of enterprises, expanded the value chain of enterprises, and enabled value co-creation among stakeholders within the ecosystem [54]. Moore [55] defined a business ecosystem as an organic business entity consisting of a series of interrelated organizations. Li and Ma [56] proposed a cross-border e-commerce ecosystem for agricultural products based on the DICE model. Wang and Ding [57] proposed that the agricultural product e-commerce ecosystem consists of dominant, key, supportive, and parasitic populations.

The concept of a platform, as a construct closely integrated with industrial practices, has been interpreted differently by scholars in different contexts. Wheelwright and Clark [58] first introduced the concept of a “platform” and pointed out that a platform can add, replace, or eliminate certain functions based on consumer needs, gradually improving the production process of products. With the rapid development of information technologies such as big data and artificial intelligence, platform enterprises are restructuring the ways of creating value, the people involved in value creation, and the places where value is obtained [59], resulting in a richer understanding of the concept of a platform. Xu [60] believes that “a platform is essentially a transaction space or place that can exist in the physical world or virtual network space. This space guides or facilitates transactions between two or more parties and strives to attract transaction participants to use the space or place by charging fees, thus pursuing maximized benefits.” Platform economics, centered on this concept, emphasizes that platform enterprises have advantages in economies of scale, scope, and network externalities, as well as access to abundant information on market participants, transactions, settlements, etc. Furthermore, scholars have constructed business ecosystems centered on platform enterprises, arguing that core platform enterprises, relying on their platform leadership, can connect innovators and transaction participants, blur the boundaries of traditional industries, integrate internal and external resources, and freely allocate production factors to enable large-scale collaborative production [49]. They also gradually evolve from internal product platforms to supply chain platforms and industry platforms, spanning multiple levels [61]. As intermediaries and data centers, platforms, together with numerous suppliers, producers, manufacturers, consumers, etc., co-create a symbiotic business ecosystem [62]. Tee and Gawer [63] aggregate the entities in an ecosystem based on their roles into demand-side systems, complementary systems, and supply-side systems, with the platform enterprise as the core providing the platform architecture, rules, and components for the interaction and innovation of species such as suppliers, demanders, and third-party complementors.

A platform ecosystem is a new organizational form based on platforms and dependent on platform-enabled interactions and transactions among businesses within the ecosystem and users. The interaction among subsystems at different levels, as well as between subsystems and the external environment, affects the overall development of the ecosystem. This is consistent with the results of the concept definition mentioned earlier, providing a framework for integrating and understanding the components and influencing factors of an open, complex, and giant system. Unfortunately, existing literature on platform ecosystems has not fully explained which influencing factors will change when complex systems are reshaped under new models and what mechanisms will affect the objectives of the entities. To address this gap, we conducted a qualitative grounded study focusing on the sustainable supply chain of cross-border live-streaming e-commerce for agricultural products in China.

## 3. Methodology

The choice of research methodology should align with the nature and content of the phenomenon under investigation. Our research question involves dynamic supply chain interactions and behaviors, addressing a relatively understudied topic in the current literature. Therefore, an exploratory theory-building approach is needed. When exploring uncharted territories and complex phenomena, it is recommended to employ qualitative research methods, such as grounded theory, to generate depth and understanding [64,65]. Grounded theory construction is also a recommended approach for theory-building in problematic and dynamic social processes [66]. Hence, we utilize programmatic grounded theory for our further research. To alleviate common criticisms of qualitative work, we carefully consider our methodology, ensuring transparency in our process and aiming to maintain a clear chain of evidence throughout the entire research process [67].

### 3.1. Data Collection

The qualitative data collection took place over a period of four months starting from October 2022. The collection process was guided by theoretical sampling criteria, and the researchers simultaneously collected, coded, and analyzed the data to develop emergent theories [68]. Initially, the sampling focused primarily on the field of live-streaming e-commerce to understand and explore the composition and influencing factors of the supply chain in live-streaming e-commerce. However, during the process of grounded theory research, we realized that the stories depicted in the materials reflected the complexity of the supply chain composition in live-streaming e-commerce. It is a complex system involving multiple stakeholders and factors. Particularly, after defining the concept of “sustainable supply chain of cross-border agri-food products in the context of live-streaming e-commerce,” we recognized the need to incorporate more analytical units. Therefore, we systematically collected materials related to keywords such as “live-streaming e-commerce,” “cross-border agri-food trade,” and “modern supply chain,” including domestic and international literature, policy texts, relevant proposals, research reports, and news cases (from 27 September 2016 to 11 January 2023). The collected raw materials amounted to over 580,000 words in written form. Data collection ceased when saturation was reached in the research findings [69].

The final samples included: (1) Content compilation of proposals related to cross-border e-commerce during the National People’s Congress and the Chinese People’s Political Consultative Conference in 2021 and 2022. (2) Research reports on national-level cross-border economic and trade exchange conferences, such as the China International Import Expo and China-ASEAN Expo. (3) Journal articles, news, and authoritative websites (such as China E-commerce Research Center, Development Research Center of the State Council, Belt and Road Research and Decision-making Platform), as well as research reports from well-known domestic and international third-party consulting agencies (such as McKinsey, Forward Industry Research Institute, iResearch). (4) Policy documents from various ministries and government departments at different levels. (5) Typical cases of cross-border agri-food e-commerce released by the Ministry of Agriculture and Rural Affairs in 2021 and 2022.

The reasons for the above sampling are as follows: (1) Cross-border e-commerce and live-streaming e-commerce are hot topics and have appeared in important documents such as government work reports and proposals for several consecutive years. There is a wealth of research reports, policy documents, and project news available, which are highly relevant and timely. Moreover, all the cases are e-commerce enterprises officially recognized by the government, with a history of at least three years, and they are already embedded or in the process of embedding in the cross-border live-streaming e-commerce supply chain, providing abundant and authentic primary data for studying supply chain influencing factors. These materials also assist in our sampling and analysis and support our theories [70]. (2) In the absence of readily available databases, many scholars in the field of supply chain management have conducted qualitative research based on similar types of materials [71]. (3) The aforementioned materials are highly relevant to the research topic. For example, in expos, live-streaming e-commerce is an important means of showcasing products to domestic and international buyers in real-time. The research reports and news articles are highly aligned with our research direction and can provide rich, original information. Policy documents are also an important source of grounded theory materials, as analyzing and interpreting them can reveal hidden insights [72].

### 3.2. Data Coding and Analysis

Data analysis was conducted in three steps: open coding, axial coding, and selective coding [46]. To ensure the systematic rigor of the analysis process, we formed a coding team consisting of multiple members, including professors, master’s and doctoral students, and industry professionals (7 individuals). On one hand, convergent coding results obtained from multiple researchers contribute to the reliability and validity of concepts and categories. Convergent viewpoints enhance the credibility of conclusions while contrasting viewpoints help avoid prematurely ending the investigation. On the other hand, the insights from team members also provide the potential for creative research, as their perspectives often complement each other, enriching the data and increasing the likelihood of extracting new insights from the data [73]. Each author independently followed coding techniques, assisted by Nvivo 11.0 software for coding. The analysis involved scanning paragraphs and regressing to words, phrases, sentences, and paragraphs to discover variables by identifying similarities and differences [74]. As data collection and analysis progressed over time, themes were modified accordingly. This led to “elevation” of participants’ responses to themes, categories, their attributes, and the scope of dimensions into an overarching theoretical explanatory scheme, ultimately resulting in the formation of new concepts or theories [66,69].

#### 3.2.1. Open Coding

Open coding primarily involves concept extraction and the initial categorization of primary categories. During this process, this study eliminated ineffective original concepts that had a repetition frequency of less than three times or were mutually contradictory. Through progressive logic, higher-level concepts and categories were extracted. This study randomly selected 2/3 of the original data for coding analysis, resulting in 48 concepts (labeled as “a+” followed by a number) and 17 categories (labeled as “A+” followed by a number) after categorization and refinement. To avoid extreme results of concept redundancy or insufficiency, each category included 2-4 concepts and original statements. The specific process of concept categorization is shown in Table 2. Table 2 only lists three categories: industrial integration, industrial development, and marketing diversion methods. Other categories were derived through the same process and logic.

#### 3.2.2. Principal Axis Encoding

Axis Coding primarily focuses on classifying the categories derived from the previous article based on their generic and correlation relationships. It aims to link categories that share similar influencing factors and abstract them into higher-level main categories. By applying Axis Coding, six main categories can be identified, as presented in Table 3.

#### 3.2.3. Selective Encoding

**Figure 2:**
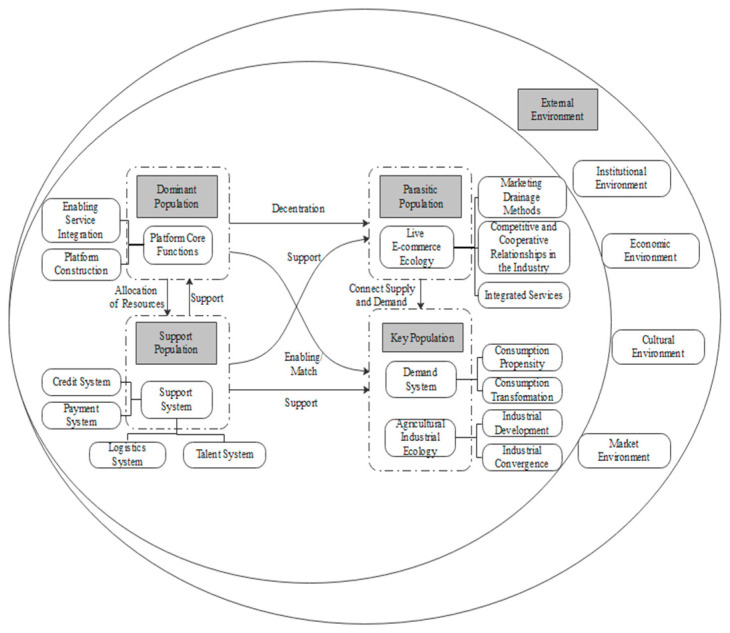
The Impact Factor System of Sustainable Supply Chain Development for Cross-border Agri-Food Products in the Context of Live-Streaming E-Commerce.

Selective Coding involves summarizing and abstracting the main categories to derive core categories that encompass all the categories. It aims to establish connections between the core categories, main categories, and other categories. Based on the inherent relationships and logic among the categories, this study has extracted the core category of “Sustainable Supply Chain of Cross-Border Agri-Food Products in the Context of Live-Streaming E-Commerce.” Building upon this core category, we sought theoretical perspectives to further explain our findings. We reviewed existing literature on ecosystem theory, platform theory, and other relevant sources, which helped refine our concepts, themes, and models. While the coding categories are derived from the original data, many insights from the literature have been discussed in relation to the concepts, offering theoretical explanations [75]. These insights were integrated into our findings. For instance, platform enterprises are recognized as the core of the system, providing platform architectures, rules, and components for the interaction and innovation of other entities [63]. Finally, this study incorporates the composition and influencing factors of the supply ecosystem into a unified analytical framework, linking the main categories and constructing an influence factor system for the development of the sustainable supply chain of cross-border agri-food products in the context of live-streaming e-commerce, as illustrated in Figure 2.

**Figure 3:**
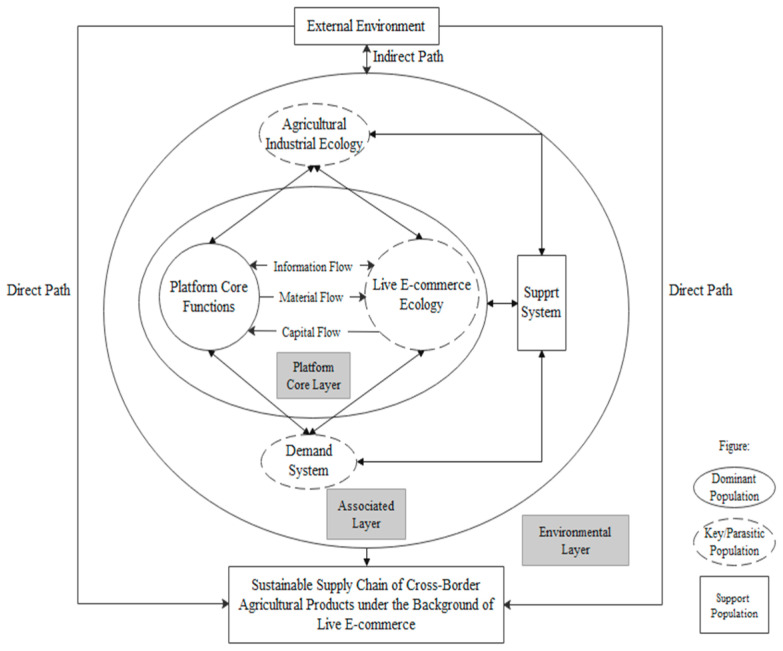
The Impact Factor Model of Sustainable Supply Chain Development for Cross-border Agri-Food Products in the Context of Live-Streaming E-Commerce.

Furthermore, the study explores the interconnections among the influencing factors and the mechanisms through which these factors impact the system’s objectives. Ultimately, an influence factor model for the development of the sustainable supply chain of cross-border agri-food products in the context of live-streaming e-commerce is constructed, as depicted in Figure 3. The formation and development of a sustainable supply chain are influenced by both internal and external factors, which can be categorized into six factors: external environment, agricultural industry ecology, core functions of the platform, live-streaming e-commerce ecosystem, demand system, and support system. These factors collectively shape the development of the sustainable supply chain.

#### 3.2.4. Theoretical Saturation Testing

The test of theoretical saturation is the fourth stage in implementing the programmatic grounded theory coding procedure. It is considered to be achieved when no new significant concepts, categories, or relationships emerge from the theoretical analysis results [76]. Coding of the remaining 1/3 of the reserved case data revealed no new important concepts, categories, or relationships, indicating a good level of theoretical saturation in the influence factor system.

Next, we evaluated our theory based on the applicability criteria of grounded theory and concluded that it meets those criteria [66,68,77,78,79].

Lastly, we assessed the reliability among the coders to ensure consistency in their coding decisions throughout the three stages of data coding. We used the “percent agreement” method among the coders, which reports the percentage of coding decisions agreed upon from the total coding decisions [80,81]. This was done in two stages. In the first round of testing, the reliability was 93%, indicating a strong level of reliability [81]. Then, the coders engaged in multiple rounds of discussion, both online and offline, to address and clarify any discrepancies in their coding [82]. The discussions regarding coding discrepancies ultimately led to a consensus among all researchers in all coding categories, resulting in 100% reliability among the raters, as shown in Table 4.

## 4. Model Explanation and Discussion

### 4.1. Model Explanation

According to the aforementioned impact factor model, the establishment and development of sustainable supply chains for cross-border agri-food products in the context of live-streaming e-commerce are influenced by both internal and external factors. The macro and micro environments of the supply chain ecosystem, including institutional, economic, cultural, and market factors, constitute its external influencing factors. These factors directly impact the construction of cross-border sustainable supply chains in live-streaming e-commerce and indirectly drive the development of the supply chain through interactions within the population of the supply chain ecosystem. On the other hand, the internal influencing factors consist of core platform functionalities, live-streaming e-commerce ecosystems, agri-food industry ecosystems, demand systems, and support systems. This finding confirms Moore’s innovation ecosystem theory, which states that an innovation ecosystem is a loosely coupled network of entities, including enterprises, that collectively develop around shared technologies, knowledge, products, and services [10]. The interactions between external environmental changes and system entities can influence the development of innovation ecosystems [85]. The following will provide specific explanations for each dimension of the model.

#### 4.1.1. Core Layer of the Platform

The core functionalities of the platform and the ecosystem of live-streaming e-commerce constitute the influencing factors of the core layer of the platform. In addition to attracting more members and elements to join the platform through self-construction, the core e-commerce platform also needs to adapt to changes in consumer attitudes and shopping patterns. It actively develops the ecosystem of live-streaming e-commerce as a channel for decentralized marketing, promotion of agricultural product brands, and real-time insights into multilevel consumer demands. It maintains value connections through the flow of information, material resources, and financial resources.

#### 4.1.2. Core Functionalities of a Platform

The impact of the core functionalities of a platform on the development of a sustainable supply chain is mainly reflected in two categories: platform construction and enabling service integration. Firstly, the platform serves as a basic service provider for other ecosystem entities, including communication mechanisms [49], transaction matching [86], and rule-making [87]. The richer the trading connections established by the virtual trading platform, the higher the value generated within the entire supply chain ecosystem [86]. Factors such as platform reputation, autonomy, and degree of decentralization also influence the user base of the platform to a certain extent. Secondly, the core platform enterprise is responsible for integrating resources and empowering the entire chain, creating performance and value for ecosystem members. On the one hand, as a gathering place for data assets, the platform can integrate and share real-time and online data and information from various modules such as production, processing, circulation, and trade and provide support for decision support systems. On the other hand, by integrating support elements within the supply chain ecosystem, the platform can provide socialized, intelligent, and modular matching services for various entities in the supply chain, thus driving the development of the supply chain ecosystem in an empowering manner [88]. For example, Alibaba, China’s largest digital agriculture platform, has proposed the “US$1000 per mu” plan and has been empowering rural agriculture in China through digital technology for many years, gradually building a comprehensive agricultural industry chain service system and achieving upgrades in six aspects: industry, products, marketing, technology, talents, and globalization. By 2020, it has established 1000 digital agricultural bases nationwide, helping Chinese farmers sell agricultural products worth over 540 billion yuan globally. This demonstrates the leading role played by the two sub-categories of platform core functionalities, which are crucial for the formation of a sustainable supply chain.

#### 4.1.3. The Live-Streaming E-Commerce Ecosystem

The impact of the live-streaming e-commerce ecosystem on the development of sustainable supply chains is mainly reflected in three domains: marketing and traffic acquisition, industry competition and cooperation, and comprehensive services. In terms of marketing and traffic acquisition, the live-streaming e-commerce model relies on two main approaches for traffic acquisition: public domain traffic acquisition (attracting traffic from content platforms, social platforms, shopping guide platforms, etc.) and private domain traffic cultivation (generating traffic increment through community expansion) [89]. Furthermore, the integration of online and offline channels and the diversification of marketing scenarios help businesses overcome temporal and spatial limitations, enabling two-way interaction with consumers and enhancing the effectiveness of traffic empowerment. Therefore, diversified and targeted marketing and traffic acquisition strategies are crucial for live-streaming e-commerce entities to enter overseas markets, as they can maximize their advantages in reducing transaction costs and rapidly matching supply and demand, ultimately achieving the goal of on-demand production [90].

In terms of industry competition and cooperation, with the popularity of live-streaming e-commerce in China, the model has successfully expanded internationally and become a hot trend in international trade [30]. Within this model, a more convenient path for cross-industry cooperation is established among members of the supply chain ecosystem, further bridging the gap between supply and demand. From the perspective of symbiotic theory, cooperative symbiosis not only expands benefits but also promotes long-term decision-making, cooperative competition, and sustainable development trends [91]. Additionally, as promotional channels expand and the effectiveness of agricultural product brands in overseas markets is enhanced, competition among different platforms and different business entities intensifies. Therefore, industry competition and cooperation have profound implications for the healthy and positive development of sustainable supply chains.

In terms of comprehensive services, the rapid growth of the live-streaming e-commerce industry has also exposed non-standard practices related to false marketing, such as selling inferior products as premium ones and engaging in malicious order brushing. E-commerce platforms and live-streaming institutions, as maintainers of the supply chain ecosystem, should ensure fairness in resolving transaction disputes, supervise the behavior of multiple stakeholders within the ecosystem, and establish mechanisms for breach of contract [92]. Moreover, the strong social nature of the live-streaming e-commerce ecosystem emphasizes community interaction and emotional value. In addition to enhancing product quality, attention should be given to exploring consumers’ spiritual demands and cultivating private domain traffic through continuous interaction. It is evident that comprehensive services play a facilitative role in the development of sustainable supply chains.

#### 4.1.4. Association Layer

The agriculture industry ecosystem, demand system, and support system constitute the influencing factors within the association layer. The platform’s core layer, with data as a crucial production factor, has the capability to continuously and automatically integrate, deconstruct, analyze, and share data from various units within the association layer. In the context of live-streaming e-commerce channels, the platform’s core layer facilitates the precise alignment of high-quality agricultural products and services with diverse consumer demands. Conversely, based on real demand, it generates the impetus for the high-quality development of the agriculture industry.

#### 4.1.5. Demand System

The impact of the demand system on the development of sustainable supply chains is primarily reflected in the categories of consumer transformation and consumer preferences. In terms of consumer transformation, driven and influenced by the COVID-19 pandemic, major e-commerce companies have implemented mobile strategies, while offline retailers have accelerated their efforts to develop online sales channels. This has led to the gradual adoption of online shopping habits by global consumers. Furthermore, their consumption preferences have exhibited new characteristics, such as reduced price sensitivity and a greater emphasis on emotional factors in consumption decision-making [93]. These characteristics have significant implications for the strategic choices and product pricing in the operations of the live-streaming e-commerce ecosystem. Moreover, consumer preferences guide the entire process of “research, production, and marketing”. Practitioners within the ecosystem need to select appropriate markets and match corresponding products based on their own positioning. Therefore, the demand system plays a crucial driving role in the development of sustainable supply chains.

#### 4.1.6. Agricultural Industry Ecology

The impact of agricultural industry ecology on the development of sustainable supply chains is mainly reflected in the aspects of industrial integration and industry development. In terms of industrial integration, the initial low value-added of agricultural products, coupled with profit segmentation among multiple supply chain nodes, makes selling products through cross-border trade channels insufficient to bring sufficient profits to the production end. The integration of primary, secondary, and tertiary industries can accelerate the penetration of advanced elements into the agricultural sector from both vertical and horizontal perspectives. This transformation allows the supply chain to evolve from single production to diversified development, thereby enhancing the quality, efficiency, and international competitiveness of the agricultural industry and achieving the goal of increasing farmers’ income [94]. Vertically, it involves the development of specialized agricultural product processing industries, focusing on improving deep processing. Horizontally, it involves innovative development of specialized agricultural functions, such as the integration of agriculture and tourism, giving rise to new models such as leisure agriculture and agri-tourism.

In terms of industry development, the “small”, “fragmented”, and “weak” characteristics of agricultural management methods have long been a major bottleneck affecting the transformation of China’s agricultural product supply chains towards modernization. Building industry clusters with leading enterprises as the core can break through the boundaries of individual companies or industries and explore the competitive advantages of specific regional agricultural product value chains from a systemic perspective. Furthermore, faced with changing market demands and the pressure of industrial transformation and upgrading, traditional agriculture lacks attention to agricultural structure, agricultural efficiency, and orderly guidance of agricultural capital. It also suffers from low technological levels, requiring breakthroughs in the current development bottleneck through agricultural modernization and upgrading. Digitalization of the agricultural industry plays a crucial role in this regard [95]. Additionally, Chinese agricultural products face challenges in aligning production standards with international standards. Different countries and regions have varying rules for cross-border e-commerce, leading to inconsistent standards for agricultural products, which prevents a significant amount of agricultural products from being directly sold through cross-border e-commerce platforms.

Finally, a large portion of Chinese agricultural products lack endorsement from regional brands [96], resulting in low recognition in the international market and difficulties in driving value output to consumers and value feedback to the production end. It is evident that the agricultural industry ecology has a significant positive effect on the development of sustainable supply chains.

#### 4.1.7. Support System

The support system, as a support population, conducts service activities centered around e-commerce platforms and ecological trading entities, enriching and improving the platform’s functionalities. The support system mainly includes credit systems, payment systems, logistics support, and talent development. In terms of the credit system, the current construction of the cross-border e-commerce market’s credit system relies mainly on the autonomy of e-commerce platforms, and its mechanisms are not yet perfect. For example, the behavior constitutions and penalty standards for illegal activities such as brush orders, push orders, and secondary sales that frequently occur during live broadcasts are difficult to clearly define solely by the platform. However, the collaborative mechanisms of government, market, and platform in innovating credit regulatory systems can provide strong guarantees for the supply chain ecology [97].

Additionally, promoting internationally recognized financial data transmission standards and achieving data standardization can ensure seamless integration between China’s cross-border payment market and the global economy, meeting the strong demand of foreign trade enterprises and retail e-commerce players for third-party cross-border payments. In terms of logistics support, cost-effective logistics solutions are crucial for perishable agricultural products. With the supply market showing a trend of resource integration and personalized customization, logistics service providers are gradually developing towards comprehensive and integrated services [98].

Moreover, supply chain professionals, as the primary resource, are key drivers of sustainable supply chain development. Building talent selection and training systems from macro, meso, and micro perspectives at the national, industry, and enterprise levels plays an important role in enhancing the vitality of the supply chain ecology and unleashing innovative skills. Through the aggregation and coordination of the core platform, the support population can form mutually supportive and functionally complementary modules, leading to architectural innovation at the ecological level of sustainable supply chains [99].

#### 4.1.8. The Environmental Layer

The environmental layer in the context of the sustainable supply chain of cross-border live-streaming agricultural products is influenced by external environmental factors, which mainly refer to the macro, meso, and micro environments in which the ecosystem operates. These external environmental factors can be categorized into four domains: institutional, cultural, economic, and market factors. Previous quantitative studies have mostly focused on examining the significance of external environmental factors for sustainable supply chains [100,101,102], but there is limited research on how these factors specifically impact the development of sustainable supply chains.

In the institutional environment, China’s trade potential has been further unleashed in recent years under the influence of strategies such as the Belt and Road Initiative and the Regional Comprehensive Economic Partnership. Apart from deepening cooperation and opening markets with other countries, China has adopted a clear strategy of national intervention in the formulation and alignment of important standards and procedures in industries such as agriculture and e-commerce. Particularly, during the emergence of new forms and models of cross-border e-commerce and live-streaming e-commerce, a small number of practitioners faced challenges in ensuring the legality and compliance of trade. In such situations, the institutional environment becomes a critical factor influencing the sustainable development of the supply chain [103].

Furthermore, in the market environment, the COVID-19 pandemic has severely impacted global industrial and supply chains, and some countries have implemented more concealed and flexible technical trade barriers for agricultural products. These barriers have significantly raised the market access standards for agricultural product exports, becoming obstacles to the free flow of agricultural products and even affecting the sustainable development of the entire agricultural product supply chain [104]. In the economic environment, as regional countries engage in orderly economic and trade cooperation, platforms for regional trade cooperation focusing on region-specific agricultural products continue to emerge, creating more trading opportunities for enterprises on both sides. For example, Chile launched the e-commerce platform “ChileB2B” in 2019, connecting Chilean exporters with importers from around the world. The platform currently has over 1000 Chilean exporters and nearly 900 buyers of goods and services.

In the cultural environment, the live-streaming e-commerce model emphasizes reconstructing endorsement mechanisms and supply-demand matching through emotional communication between people. This highlights the importance of cultural attributes in cross-cultural communication within the context of cross-border live-streaming e-commerce [105]. Merely replicating successful domestic experiences can result in a lack of adaptation to local cultures. It is essential to fully consider the impact of cultural heterogeneity on business operations and integrate local cultural symbols to create differentiated characteristics, thereby achieving the upgrade from product export to brand export and cultural export. Overall, the macro and micro environments directly impact the development of sustainable supply chains and further promote the development of sustainable supply chains through the mutual interactions between the internal population of the cross-border live-streaming agricultural product supply chain ecosystem.

### 4.2. Discussion

Our research, for the first time, employs qualitative research methods to explain the factors and mechanisms that influence the development of China’s cross-border live-streaming e-commerce sustainable supply chain. We found that the development of this supply chain is influenced by a combination of internal and external factors, which can be further classified into six factors. Moreover, we have developed a theoretical framework to explain the process of their interactions.

This model consists of two driving paths corresponding to different mechanisms under the influence of internal and external factors. Importantly, this conclusion is supported and integrated by the ecosystem theory. While some of our findings align with current literature, others expand and deepen a new subfield, filling the theoretical gap in the integration of the live-streaming e-commerce ecosystem and the cross-border agricultural supply chain development model and constructing a systematic research framework. Furthermore, this rooted model also covers a broader research scope, extending beyond the current scenario and applying to other physical fields, leading to higher and more universal theories [106].

#### 4.2.1. Theoretical Implications

Existing research has made valuable explorations into traditional agricultural supply chains and their extensions, including the conceptual connotations and influencing factors. Many studies have focused on the overall level of supply chains [43,107,108]. However, in the case of emerging live-streaming e-commerce models, they are often treated as independent business models, as if the factors influencing their development are limited to the live-streaming e-commerce ecosystem (such as anchors, users, platforms, etc.) [6,10], without fully considering how live-streaming e-commerce is increasingly integrating into the value chains of other industries, thereby changing their positions and value capture [109]. For example, in the agriculture and cross-border trade fields, the empowering role of live-streaming e-commerce can reconstruct the cross-border agricultural supply chain. In this open and complex system, changes in various links (such as quality, pricing, etc.) have impacts on the supply chain entities [90,110]. Our research fills this theoretical and research framework gap for this new form of foreign trade and expands the study of sustainable supply chain management in terms of industry- and location-specific factors [35,111].

Ecosystem theory has previously been introduced by researchers into the field of business studies, investigating the forms, compositional structures, functional characteristics, and interaction relationships of internal enterprises, enterprise communities, and business ecosystems [112,113,114]. Correspondingly, platform theory has been applied to study the roles and functions generated by platforms as important organizational forms in industrial practices [49,62]. However, bridging the two theoretical perspectives is limited to studying the compositional structures and interaction relationships of business ecosystems where platform enterprises are located [61,62] without accurately explaining which influencing factors will change and what mechanisms will affect the goals of the entities when open and complex systems reshape across industries and supply chains. In the current study, we introduced the live-streaming e-commerce model into the cross-border agricultural supply chain and found that some critical internet enterprises in the live-streaming e-commerce field are not just websites or multinational corporations that provide transaction matching services [86]. They are platforms that connect a large number of users/customers with service providers, advertisers, or other users, organizing, reorganizing, and even reforming many industries [115,116]. From this, we expanded the application of platform ecosystem theory to explain the factors and mechanisms of China’s cross-border live-streaming e-commerce sustainable supply chain development, which is centered around platform enterprises.

In addition, in our research, the live-streaming e-commerce ecosystem is a dependent population that relies on core platform enterprises [117] and serves as an important channel to link consumer groups and meet their multidimensional and multilevel needs. Core platform enterprises actively develop the live-streaming e-commerce ecosystem to adapt to changes in consumer attitudes and shopping patterns, which also restructure the economic structure of the supply ecosystem. Participation in these ecosystems is crucial for the survival and growth of many enterprises [115,118]. Moreover, as core platform enterprises possess data resources, which are key production elements for the entire ecosystem, they can organize various economic (as well as political and social) activities [119] by arranging them in socialized, intelligent, and modular supply-demand matching [88], such as the matching of service resources between the support system and the agricultural industry ecosystem, and the matching of service and product resources between the demand system and the agricultural industry ecosystem. We believe that this model can go beyond the live-streaming e-commerce ecosystem and be applied to research the adaptability to external environmental changes and the inclusiveness of the food and nutrition system, which is centered around data-intelligent platforms, but further evidence is required.

#### 4.2.2. Implications for Practice

**Figure 4:**
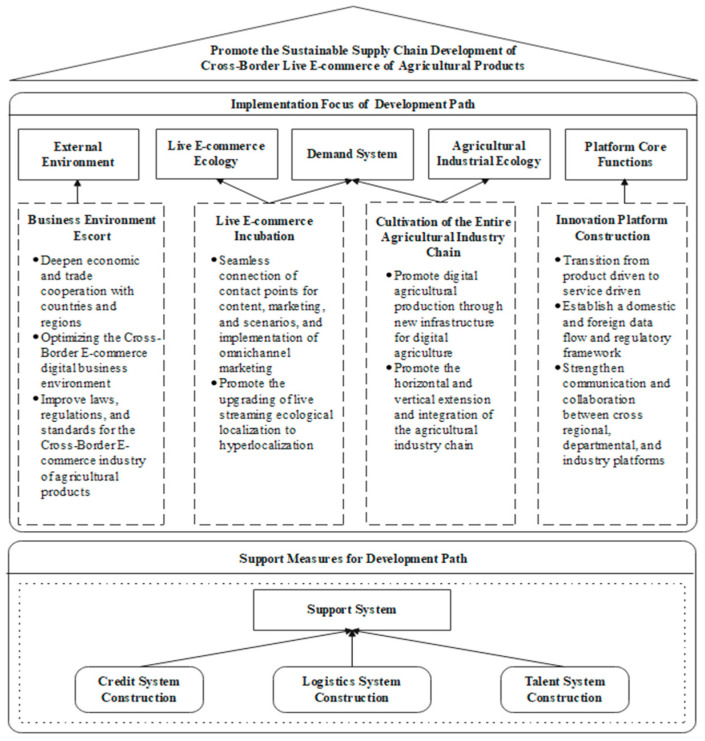
Framework Diagram of Sustainable Supply Chain Development Path for Cross-Border Agri-Food Products in the Context of Live-Streaming e-Commerce.

Based on the analysis and comprehensive consideration of China’s national conditions and industrial development status, from the perspectives of relevant practitioners, government departments, and organizations, we propose a development path for the sustainable supply chain of cross-border agricultural products under the background of live-streaming e-commerce. This path involves fully leveraging the guiding role of market demand, relying on core e-commerce platform enterprises in the agriculture and foreign trade fields, utilizing live-streaming e-commerce as a distribution channel, strengthening the planning, guidance, and coordination of the government and relevant industry organizations, establishing a sound support system for cross-border live-streaming e-commerce of agricultural products, and promoting mutual benefits, shared development, and collaborative development among all members. This will create a well-structured and highly efficient operational mechanism for the supply chain ecosystem. The framework diagram of the development path is shown in Figure 4.

1. Key Implementation Priorities of Development Path

(1) Safeguarding the Business Environment

Optimizing the business environment is a crucial foundation for ensuring the rapid and healthy development of cross-border agricultural product supply chains in live streaming. Safeguarding the business environment should focus on the following three aspects. The first is deepening economic and trade cooperation with national and regional partners. It includes organizing trade exchange platforms, such as expos, shopping festivals, and trade fairs, targeting different regions, industries, and entities to provide convenient platforms and channels for enterprises to access and integrate into international markets. The second is optimizing the digital business environment for cross-border e-commerce. It encompasses seizing the opportunity of constructing pilot free trade zones, bonded areas, and cross-border e-commerce pilot zones and innovating management and services in market access, customs clearance and quarantine, payment, and credit to explore replicable and scalable experiences. Last is improving the legal regulations and standards for the cross-border e-commerce industry of agricultural products, participating actively in the formulation of international standards and rules for cross-border e-commerce of agricultural products, and referring to internationally recognized food quality management standards, such as ISO9001 [120] and HACCP, to continuously improve the legal regulations and technical standards system applicable to the cross-border agricultural product supply chain.

(2) Innovation Platform Construction

Currently, China’s cross-border e-commerce ecosystem consists of numerous small-scale enterprises, lacking overall standardization. It is necessary to promote the development of core comprehensive platforms that have information advantages and can integrate and empower services. Firstly, we need a shift from a product-driven approach to a service-driven approach. We need to establish platforms that provide data analysis and decision-making services, such as market insights, marketing analysis, and intelligent pricing, to the entire supply chain, driving the development of the industry ecosystem in a holistic manner. Secondly, we need to establish a domestic and international data flow regulation framework. We need to encourage the sharing and openness of public data while establishing a flexible multilateral privacy and data protection regulatory cooperation system, enabling a barrier-free flow of personal information based on protection. Thirdly, we need to strengthen communication and collaboration among comprehensive platforms across regions, departments, and industries. We need to utilize digital technologies, such as the Internet of Things (IoT) and industrial internet, to achieve business collaboration in various stages of the supply chain, including raw material procurement, production and manufacturing, and end sales, creating an industry collaboration platform that facilitates smooth international expansion of brands and enterprises.

(3) Live-streaming e-commerce Incubation

Live-streaming e-commerce is still a nascent phenomenon in overseas markets, and the operational logic in China can be partially applied to overseas live-streaming e-commerce models. However, differences in social preferences, shopping habits, cultural customs, and other aspects among consumer groups require practitioners to make localized adjustments. Firstly, we should seamlessly connect the touchpoints of content, marketing, and scenarios, implementing omni-channel marketing. Live-streaming e-commerce emphasizes continuous multi-sensory interaction and perception of consumer needs, bridging the elements of interpersonal communication with the convenience of e-commerce. Secondly, we should promote the localization of live-streaming ecosystems towards super-localization. Despite globalization, ideological differences and cultural boundaries still exist. During live streaming, practitioners need to introduce localized product design, production, and marketing promotion methods based on the local cultural background. Construct feedback mechanisms between design, production, and consumer experiences, opinion leaders, and consumers to gain recognition and emotional resonance from consumers with different cultural backgrounds.

(4) Cultivating the Agricultural Full Industry Chain

Considering the trend of consumer upgrades in demand systems, it is necessary to actively promote the quality transformation, efficiency transformation, and dynamic transformation of the agricultural industry ecosystem to support the transformation and upgrading of the agricultural industry and achieve breakthroughs in benefits. Firstly, we need to promote digital agricultural production through new infrastructure in digital agriculture. Based on the analysis and prediction of consumer-side big data, realize standardized, automated, and precision planting on the production side. Secondly, we need to promote horizontal and vertical extension and integration of the agricultural industry chain. Highlight the development of the specialty agricultural product processing industry, focus on improving deep processing, cultivate agricultural brands, and further enhance the added value of agricultural products. Simultaneously, explore and utilize the value of agriculture in technology, tourism, and health care, cross-industry allocation of agricultural and modern industrial elements, and actively develop new industries and formats that integrate with agriculture, such as leisure agriculture and agri-tourism.

2. Supportive Measures for Development Path

(1) Strengthening the Credit System

Promoting the harmonious development of the cross-border e-commerce ecosystem requires a robust credit system. It is necessary to establish a social governance mechanism that includes government regulation, self-governance by entities, industry self-discipline, user choice, and social supervision. This will help standardize the behavior of various stakeholders involved in the ecosystem.

(2) Improving Logistics System Development

The enhancement of efficiency and benefits in cross-border e-commerce imposes higher requirements on the fulfillment capacity and service levels of e-commerce logistics. There is an urgent need to improve the digital infrastructure of logistics, such as channel networks, cargo organization, and air transportation, by promoting co-construction and sharing among regions. Simultaneously, simplifying customs clearance procedures and facilitating information sharing among customs, taxation, ports, airports, and postal services in logistics, security inspection, and customs clearance is essential.

(3) Developing Talent Systems

Innovative talent development mechanisms are needed to build a comprehensive talent cultivation system for cross-border e-commerce. This involves a combination of cultivating existing talents and attracting external talents. It is crucial to improve the talent cultivation system in related industries and encourage general and vocational institutions to offer majors or courses related to cross-border e-commerce and digital trade. This will ensure the nurturing of highly skilled and versatile professionals who meet the demands of the new foreign trade formats.

## 5. Conclusions and Future Research

### 5.1. Conclusions

This study revolves around the core question of “factors influencing the sustainable development of cross-border agricultural supply chains under the context of livestreaming e-commerce.” It addresses this question through three stages: “conceptual summarization, model construction, and path proposition.” Firstly, it clarifies relevant concepts and distills the essence and basic characteristics of this new model. Secondly, utilizing the Programmatic Rooting Theory, it constructs a system of influencing factors and an action model for the sustainable development of cross-border agricultural supply chains in the context of livestreaming. Finally, from the perspectives of government agencies and practitioners, it proposes a development path for sustainable cross-border agricultural supply chains under the context of livestreaming e-commerce. The main research conclusions are as follows:

(1) The sustainable cross-border agricultural supply chain under the context of livestreaming e-commerce emerges against the backdrop of cross-border agricultural trade development and the rise of live-streaming e-commerce. It represents a significant outcome and manifestation of the deep integration and collaborative development between the new livestreaming e-commerce model and the traditional agricultural product industry supply chain. Its fundamental features include data as a key production element, connecting consumer groups through livestreaming e-commerce channels, a systemic approach involving economics, society, environment, and resilience, with aspects of ubiquity, intelligence, and integration, ultimately achieving sustainable development of live-streaming cross-border agricultural supply chains.
(2) The development of the sustainable cross-border agricultural supply chain under the context of livestreaming e-commerce is influenced by both internal and external factors. The external environment of the supply chain ecosystem (institutional, economic, cultural, market) constitutes its external influencing factors, with two main driving paths: direct impact on the construction of live-streaming cross-border agricultural supply chains and indirect driving of supply chain development through interactions among internal population within the supply chain ecosystem. On the other hand, platform core functions, live-streaming e-commerce ecosystem, agricultural industry ecosystem, demand system, and support system collectively form its internal influencing factors.
(3) Based on the supply chain model and considering China’s national conditions and industrial development status, the implementation focus of the development path for sustainable cross-border agricultural supply chains under the context of live-streaming e-commerce includes safeguarding the business environment, innovative platform construction, live-streaming e-commerce incubation, and comprehensive agricultural industry chain cultivation. Measures to enhance credit, logistics, and talent systems are used as support guarantees, aiming to establish a logically planned and efficiently operating supply chain ecosystem operational mechanism.

### 5.2. Limitations and Future Research

There are several potential directions for future research. Firstly, the model constructed in this study is based on small-sample qualitative research, and its reliability and validity have not been tested on a large sample. Future research can empirically verify the proposed model and the generalizability of the theory constructed using the grounded theory approach. This is an important subsequent step in establishing the theory based on this study. Secondly, due to the uniqueness of the agricultural sector, there is considerable differentiation among sub-industries, and the industrial systems and supply chain conditions vary significantly among different sub-industries and even different agricultural products. Future research can conduct precise analyses based on the classification of agricultural sub-industries and products, providing more accurate optimization directions for industry practitioners. Thirdly, although this study categorizes the core functions of platforms as an important main category, the scope of this study does not address the question of whether these core functions, rooted in live-streaming e-commerce ecosystems, can be applied to a broader range of physical sectors. In particular, the formation and development of this sustainable supply chain are influenced by both internal and external factors. Future research can consider applying this model to the research on the adaptability to external environmental changes and the inclusiveness of a nutrition system dominated by data-intelligent platforms. Fourthly, future research can investigate the development of cross-border live-streaming e-commerce in Southeast Asia and other geographical regions. The samples in this study include data materials related to global trade, but the relevant policy texts and proposals are mainly from China. Differences in industry status, government regulation, and infrastructure among different regions can have an impact on the development of sustainable supply chains.

## Figures and Tables

**Table 1:** The Evolution of the Concept, Connotations, and Characteristics of Sustainable Supply Chains for Cross-Border Agri-Food Products in the Context of Live-Streaming E-Commerce.

Concept	Connotations	Characteristics
Agri-food Products Supply Chain	Based on the core agricultural enterprises, the sustainable supply chain for cross-border agricultural products is a networked system that connects various participants, including producers, consumers, and numerous intermediary entities. This system effectively controls the flow of funds, materials, and information, enabling smooth and value-added processes.	(1) Seasonality of production (2) Traceability (3) Strong regional and cultural dependencies (4) Dual uncertainty of supply and demand
Cross-border Agri-food Products Supply Chain	A cross-border supply chain network is formed by leveraging core agricultural enterprises involved in agriculture and foreign trade in different countries or regions. This network covers the entire process of agricultural production, processing, distribution, and trade. It operates through the flow of information, materials, and funds, facilitating the seamless coordination and integration of various stages in the supply chain.	(1) A system engineering approach constrained by multiple stakeholders and factors (2) Transnational movement of elements (3) Challenges in quality and safety traceability (4) Multilevel, cross-functional, and complex hierarchical structure
The Sustainable Supply Chain of Agri-food Products	A supply chain system based on digital information technology and led by the core enterprises is established by integrating and coordinating various elements of each node in the entire supply chain. Its purpose is to achieve sustainable and coordinated development in the economic, social, and environmental dimensions and enhance the overall resilience of the supply chain to internal and external risks.	(1) Collaboration (2) Integration of Informationized Production Factors (3) Inclusion of Economic, Social, Environmental, and Resilience Aspects (4) Shared Benefits and Risk Sharing among Members
The Sustainable Supply Chain of Cross-border Agri-food Products in the Context of Live-streaming E-commerce	The supply chain ecosystem, formed with the support of core enterprises involved in agriculture and foreign trade, leverages modern information technology to enable distribution through live-streaming e-commerce channels, driven by authentic demand and supported by socialized services. This ecosystem, governed by the control of financial flow, material flow, and information flow, enables the maximization of overall supply chain efficiency and multidimensional sustainable coordination and development.	(1) With data as a crucial production factor (2) Linking consumer groups through live-streaming e-commerce channels (3) It encompasses a systemic engineering approach involving economic, social, environmental, and resilience aspects (4) Characterized by ubiquity, intelligence, and integration.

**Table 2:** Open Coding in the Realm of Industrial Integration, Industry Development, and Marketing Diversion Methods.

Original Statement	Conceptualization	Categorize
The company has invested over one million yuan to establish a new production and processing line, making it the only vinegar pepper processing factory in Batang with SC quality certification.	Integration of the Primary and Secondary Industries (a1)	Industrial Integration (A1)
Yunfu City showcases the achievements of rural revitalization and the new development of rural tourism by hosting the Food Festival (Sweet Potato Festival) in conjunction with the Harvest Festival, highlighting the implementation of rural revitalization efforts.	Integration of the Primary and Tertiary Industries (a2)	
The city has successfully completed the construction of two demonstration zones for apple and grape production, and various agricultural production bases have established an integrated supply chain system that covers the entire process from production to supply and marketing for agricultural products.	Industrial Clusters (a3)	Industrial Development (A2)
Deqing County has collaborated with Top Cloud Farm and JD.com to establish a smart agriculture demonstration site for early bamboo shoots, aiming to create an integrated information platform for the entire early bamboo shoots industry chain. The platform incorporates smart production, remote monitoring, scientific management, and other functionalities.	Industrial Transformation and Upgrading (a4)	
Chinese agricultural products face challenges such as the lack of alignment between production standards and international standards. Additionally, different rules and regulations of cross-border e-commerce platforms in various countries and regions create obstacles, resulting in many agricultural products being unable to be directly sold through cross-border e-commerce platforms.	Standardization Construction (a5)	
Efforts are being made to build a regional brand, “Chagan Lake,” known for its green and organic products. The focus is on developing e-commerce direct sales and leveraging the popularity of live-streaming platforms to promote and sell these products.	Brand Cultivation (a6)	
Promoting Thai coconut products on Chinese social media platforms, including organizing promotional activities during important Chinese festivals, to capture the attention of consumers and generate interest in the products.	Social Media Marketing (a7)	Marketing Funnel Strategies (A3)
The Winter Fair is an important platform for showcasing Hainan’s rural development achievements and effectively connecting poverty alleviation efforts with rural revitalization. It plays a significant role in promoting agricultural exchange and cooperation between the Hainan Free Trade Port and countries and regions along the Belt and Road Initiative.	Outbound Marketing (a8)	
The Foreign Affairs Office of Nanning City, Guangxi, together with the Consulates General of Malaysia, Thailand, and Vietnam in Nanning, jointly organized the “Consuls General Online Live Streaming Sales Event” during the month of March.	Influencer Marketing (a9)	
The third China International Import Expo (CIIE) pays more attention to the integration of online and offline activities, actively exploring the “cloud-based” exhibition and participation model.	Online and Offline Integration (a10)	

**Table 3:** Axial Coding.

Main Category	Corresponding Category	Connotations of Category
Agricultural Industry Ecosystem	Industrial Integration	The degree of integration between the supply-side primary, secondary, and tertiary industries affects the operation of the supply system.
	Industrial Development	The level of industrial development in terms of supply-side industry clustering, transformation and upgrading, standardization construction, and brand cultivation affects the operation of the supply system.
Core Functionalities of the Platform	Integrated Empowerment Services	The performance of platform core functions is influenced by the functionality of cross-border e-commerce service platforms along the supply chain, the establishment of digital agricultural support systems, and the availability of data support throughout the entire process.
	Platform Development	The performance of platform core functions is influenced by the platform’s reputation, autonomy, and decentralization.
Live E-commerce Ecosystem	Marketing and Lead Generation Strategies	The choice of marketing and traffic acquisition methods can impact the operation of the live-streaming e-commerce ecosystem.
	Industry Competition and Cooperation	Competition among cross-border live-streaming e-commerce giants and interactions between the internet, agriculture, and other industries can influence the operation of the live-streaming e-commerce ecosystem.
	Comprehensive Services	The comprehensive services provided by ecosystem participants, such as platform regulation, after-sales services, and continuous interaction, can impact the operation of the live-streaming e-commerce ecosystem.
Support System	Credit System	The soundness of the supply chain credit system can impact the support system.
	Payment System	The robustness of the supply chain payment system can affect the support system.
	Logistics Support	The level of improvement in the supply chain logistics system can impact the support system.
	Talent Development/Training	The effectiveness of supply chain talent development can impact the support system.
Demand System	Consumer Transformation	The degree of transformation in shopping patterns and consumer attitudes on the demand side can impact the demand system.
	Consumer Preferences	The demand inclination of consumers towards goods can affect the demand system.
External Environment	Institutional Environment	Government policies, regulations, and streamlined processes can influence the external environment of the live-streaming cross-border sustainable agricultural supply chain.
	Economic Environment	Economic cooperation and the level of economic development can impact the external environment of the live-streaming cross-border sustainable agricultural supply chain.
	Cultural Environment	Cultural heterogeneity, cultural identity, and religious beliefs can influence the external environment of the live-streaming cross-border sustainable agricultural supply chain.
	Market Environment	Market influence and international market barriers, among other market factors, can impact the external environment of the live-streaming cross-border sustainable agricultural supply chain.

**Table 4:** Data Trustworthiness and Methods of Assurance.

Trustworthiness Criteria	Method of Addressing Criteria in This Study
Credibility: The extent to which the results appear to represent the data	(1) Summary of background provided to coding participants for initial understanding. (2) Regular organization of team interactions and reporting. (3) Analysis of code and text conducted by independent coding personnel. (4) Independent researchers reviewed the interpretations. (5) Result: The influence factors model is continuously evolving.
Transferability: The extent to which the findings from one study in one context will apply to other contexts	(1) Theoretical sampling was conducted. (2) Result: All resource data was represented using theoretical concepts.
Dependability: The extent to which the findings are unique to time and place; the stability or consistency of explanations	(1) Strict adherence to data collection and interpretation guidelines was followed [83,84]. (2) Results: Consistency in the narratives was observed across different organizations, regardless of the position of the supply chain nodes and the timing of the events.
Confirmability: The extent to which interpretations are the result of the participants and the phenomenon as opposed to researcher biases	(1) Summaries of interpretations, documents, and preliminary findings were independently reviewed by at least three researchers. (2) Results: Interpretations were expanded and validated.
Integrity: The extent to which interpretations are influenced by misinformation or evasions by participants	(1) Multiple sources and perspectives were considered when collecting data. (2) Results: Researchers gained a deeper and more comprehensive understanding of the research subject.
Fit: The extent to which findings fit with the substantive area under investigation	(1) Methods addressing credibility, reliability, and confirmability were employed. (2) Results: Concepts were described in greater depth, theoretical integration became more fluid and less linear, capturing the complexity of social interactions identified in the data.
Generality: The extent to which findings discover multiple aspects of the phenomenon.	(1) Grounding had sufficient length and openness to allow for exploration of various complex aspects of phenomena and related concepts. (2) Results: Multiple aspects of the phenomena were captured.

## Data Availability

Not applicable.
